# 1715. Use of a validated tool to assess transition readiness to adult care of adolescents living with HIV in Rwanda

**DOI:** 10.1093/ofid/ofad500.1548

**Published:** 2023-11-27

**Authors:** Tanya Rogo, Febronie Mushimiyimana, Juliette Unyuzumutima, Lisine Tuyisenge, Rami Kantor

**Affiliations:** Brown University Alpert Medical School, Providence, Rhode Island; University Teaching Hospital of Kigali, Kigali, Kigali, Rwanda; University Teaching Hospital of Kigali, Kigali, Kigali, Rwanda; University Teaching Hospital of Kigali, Kigali, Kigali, Rwanda; Alpert Medical School of Brown University, Providence, Rhode Island

## Abstract

**Background:**

Recent African studies report that only 11-60% of transitioned adolescents living with HIV (ALHIV) were successfully retained in care after healthcare transition (HCT) from pediatric to adult care settings. HCT without adequate pre-transition preparation can result in loss of follow-up and poor treatment adherence. Transition guidelines recommend longitudinal transition readiness assessment using an objective measure, a practice not routinely implemented.

**Methods:**

During March-April 2023, HCT readiness was assessed in a cross-sectional study of Rwandan ALHIV age ≥ 18 years attending the University Teaching Hospital of Kigali pediatric HIV clinic and about to transition to adult care. Transition readiness was assessed using the validated Transition Assessment Readiness Questionnaire (TRAQ 5.0), consisting of 20 questions in five subscales: Appointment Keeping; Tracking Health Issues; Managing Medications; Talking with Providers; and Managing Daily Activities. Responses were graded on a Likert scale of 1-5 (1 being “No, I do not know how” and 5 being “Yes, I always do this when I need to”). Scores less than 4 indicate need for education.

**Results:**

In 32 perinatally-infected ALHIV, mean age at diagnosis was 3.7 years (SD 2.7) and at antiretroviral therapy initiation 5.2 years (SD 3.3). Mean number of clinic visits in the prior 12 months was 4 (S.D 0.48, range 3-6). Half were still in high school, and most (88%) lived with their biological parents. The mean TRAQ scores were Appointment Keeping 3.82 (SD 0.61); Tracking Health Issues 3.43 (SD 0.93); Managing Medications 4.56 (SD 0.59); Talking with Providers 4.71 (SD 0.4); and Managing Daily Activities 4.52 (SD 0.46).

**Conclusion:**

In a first-of-its-kind study in East Africa, a validated HCT assessment in Rwandan ALHIV suggests overall readiness in managing medications, daily living tasks and communication with providers. Needs for further education in tracking health and keeping appointments were identified, important for independent self-management of chronic disease. Longitudinal follow-up and comparison to other populations (now feasible due to using a validated tool) are needed, to develop and further evaluate the impact of needed interventions on post-transition outcomes.

**Disclosures:**

**Tanya Rogo, MD**, AstraZeneca: Grant/Research Support|Pfizer: Advisor/Consultant|Pfizer: Grant/Research Support

